# Maternal and dietary behavior-related factors associated with preterm birth in Southeastern Terai, Nepal: A cross sectional study

**DOI:** 10.3389/fpubh.2022.946657

**Published:** 2022-09-15

**Authors:** Dilaram Acharya, Salila Gautam, Thomas G. Poder, Antoine Lewin, Amaury Gaussen, Kwan Lee, Jitendra Kumar Singh

**Affiliations:** ^1^Department of Management, Evaluation and Health Policy, School of Public Health, Université de Montréal, Montréal, QC, Canada; ^2^Medical Affairs and Innovation, Héma-Québec, Montréal, QC, Canada; ^3^Independent Researcher, Montréal, QC, Canada; ^4^Centre de Recherche de l'Institut Universitaire en Santé Mentale de Montréal, CIUSSS de l'Est-de-l'île-de-Montréal, Montréal, QC, Canada; ^5^Faculty of Medicine and Health Science, Université de Sherbrooke, Sherbrooke, QC, Canada; ^6^Department of Preventive Medicine, College of Medicine, Dongguk University, Gyeongju, South Korea; ^7^Department of Community Medicine, Janaki Medical College, Tribhuvan University, Janakpur, Nepal

**Keywords:** antenatal care, cross-sectional study, dietary behavior, obstetric history, preterm birth, deprived group, Nepal

## Abstract

**Background:**

Preterm birth (PTB) is a global issue although its burden is higher in low- and middle-income countries. This study examined the risk factors of PTB in Southeastern Terai, Nepal.

**Methods:**

In this community-based cross-sectional study, a total of 305 mothers having children under the age of 6 months were selected using systematic random sampling. Data were collected by structured interviewer-administered questionnaires and maternal antenatal cards from study participants for some clinical information. Predictors of PTB were identified using multi-level logistic regression analysis at a *P*-value < 0.05.

**Results:**

Of the total 305 mother-live-born baby pairs, 13.77% (42/305) had preterm childbirth. Maternal socio-demographic factors such as mothers from Dalit caste/ethnicity [adjusted odds ratio (AOR) = 12.16, 95% CI = 2.2–64.61] and Aadibasi/Janajati caste/ethnicity (AOR = 3.83, 95% CI = 1.01–14.65), family income in the first tercile (AOR = 6.82, 95% CI = 1.65–28.08), than their counterparts, were significantly positively associated with PTB. Likewise, other maternal and dietary factors, such as birth order first-second (AOR = 9.56, 95% CI = 1.74–52.53), and birth spacing ≤ 2 years (AOR = 5.16, 95% CI = 1.62–16.42), mothers who did not consume additional meal (AOR = 9.53, 95% CI = 2.13–42.55), milk and milk products (AOR = 6.44, 95% CI = 1.56–26.51) during pregnancy, having <4 antenatal (ANC) visits (AOR = 4.29, 95% CI = 1.25–14.67), did not have intake of recommended amount of iron and folic acid tablets (IFA) (<180 tablets) (AOR = 3.46, 95% CI = 1.03–11.58), and not having adequate rest and sleep (AOR = 4.83, 95% CI = 1.01–23.30) during pregnancy had higher odds of having PTB than their counterparts.

**Conclusion:**

Some socio-demographic, maternal, and dietary behavior-related factors were independently associated with PTB. These factors should be considered while designing targeted health interventions in Nepal. In addition, we recommend specific measures such as promoting pregnant women to use available antenatal care and counseling services offered to them, as well as having an adequate diet to a level that meets their daily requirements.

## Introduction

Preterm birth (PTB) refers to a live birth that occurs before 37 completed weeks of gestation (1). Depending upon the gestational age, the preterm has been categorized as extremely preterm (<28 weeks), very preterm (28 to 32 weeks), and moderate to late preterm (32 to 37 weeks) (1, 2). Of approximately 15 million PTB occurring annually worldwide, 1 million of them die as a result of preterm-related complications before the child reaches the age of 5 years. A majority of such events occur in low- and middle-income countries, especially Southeast Asia and sub-Saharan Africa (3). More efforts are therefore necessary for low- and middle-income countries to achieve the United Nations Sustainable Development Goal 3 target 3.2 which aims to end all preventable deaths of newborns and children aged under 5 years of age by 2030 (3–5). Nepal remained with the 20^th^ highest rate of PTB in the world in 2010 (6), and prematurity has often been reported as one of the major causes of neonatal deaths in Nepal (7–9).

PTB has numerous short- and long-term impacts on children. Children with PTB have a lower chance of survival; they are more likely to contract respiratory and other common childhood infections (6, 10–12), and they have long-term neurodevelopmental impairment that leads to poor learning abilities, cognition, behavior, and reduced physical and mental ability; as well as several non-communicable health problems such as hypertension, cardiovascular and cerebrovascular diseases; type 2 diabetes; chronic kidney disease; asthma; and pulmonary abnormalities (11–13). In addition, PTB is associated with increased hospitalization rates and healthcare costs, leading to a burden on the healthcare system and economy (14–16).

Several prior scientific reports have explored a wide range of maternal socio-demographic characteristics such as younger (<20 years) or advanced maternal age (>35 years) (17–20), none or lower educational level (20–22), rural residents (20), none or suboptimal utilization of antenatal care services (17, 20, 23, 24), existing or previous maternal obstetrical and medical conditions such as the history of previous PTB, adverse birth outcomes and abortions, pregnancy-induced hypertension, preeclampsia, antepartum hemorrhage, premature membrane rupture, multiple pregnancies, short (≤ 2 years) birth spacing (20, 22–24), infectious diseases, and anemia occurring during pregnancy (24) were independently positively associated with PTB.

In Nepal, few hospitals and community-based studies have reported on the status of PTB in recent decades (25–28). In addition, little is known about the risk factors of PTB in Nepal (26, 28). A recent study from Nepal mainly examined the sociodemographic, maternal, and obstetrical factors associated with the outcome of interest (26), while another previous study reported that young maternal age (< or = 18 years) increased the risk of PTB (28). This study was designed and performed to fill the literature gap and explore some additional factors associated with PTB in Nepal. In addition, we believe that the findings of this local area research might provide evidence to modify some of the modifiable associated factors of PTB, guide with better counseling pregnant women, and help local area strategy formulation to reduce long-standing problem of PTB.

## Materials and methods

### Study design, area, and subjects

A community-based cross-sectional study was carried out in Kurtha of Janakpur sub-metropolitan city in the Dhanusa district of Southeastern Terai, Nepal. The study area consisted of several clusters located in a semi-urban area within a 3-km distance from the Janakpur sub-metropolitan city. The study area is located in the southern plains of Madhesh province, Nepal. The Madhesh province consists of eight districts, including Dhanusha. Most people in the study area are similar to people residing in the same province in terms of language, culture, and literacy status (29).

**Figure 1:**
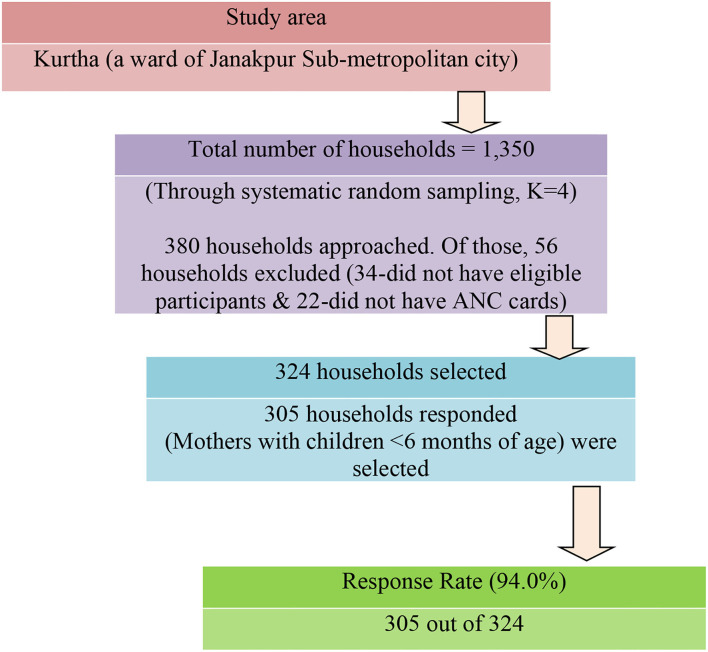
Sampling strategy.

According to the 2011 census, the study area included 1,350 households with a population of approximately 7,815 individuals. The total number of women of reproductive age (15–49 years) was 1,820 (30). The sampling frame was generated through the list of households with a list of women obtained from the record of the Sub-metropolitan city. A total of 305 households from the study area were selected using systematic random sampling. The sampling interval (K = 4) was determined by dividing the total number of households by the sample size. The first household selection followed a simple random sampling technique. Mothers with children <6 months of age in the selected households constituted the study participants. If a mother with a child <6 months of age was not found in the selected household, the subsequent households were recruited until the eligible participant requirement was met. Of a total of 380 households visited, 56 households did not meet the selection criteria (34 households did not have study participants, and 22 did not have ANC cards with them). Finally, we got responses from 305 households out of 324, giving rise to a response rate of 94% (Figure 1). Random selection (lottery method) was used in case of more than one study subject was available in the selected household.

A sample of 305 study subjects was estimated using the formula, *n* =Z^2^pq/d^2^ (31), where, *Z* = 1.96, *p* = proportion of gestational age <37 weeks in Nepal = 17.2% (32), *q* = (1–p) = (100–17.2) = 82.8%, and *d* = desirable error 0.05 (5% margin of error). Thus, we found *n* = 219 study subjects. Considering the design effect of 1.25 and the non-response rate of 10%, we required 302 study subjects. Finally, we enrolled a total of 305 study participants in this study.

### Data collection

Data were collected between 1 July 2019 and 20 August 2019. Face-to-face interviews were conducted by 10 medical undergraduate students after they participated in a 5-day data collection training. Data were collected using a structured questionnaire adapted from the Nepal Demographic and Health Survey (NDHS) 2016 (33). The outcome variable of the study was PTB. It was defined as the birth of a baby at fewer than 37 weeks gestational age or fewer than 259 days since the first day of the women's last menstrual period (LMP) (1). Gestational age was recorded from the antenatal card (community midwives maintain such records in rural Nepal) which was obtained directly from the study participants during data collection. The major independent variables of interest were nature of the diet, consumption of additional meals (including one more meal apart from the regular diet/meal by pregnant women.), consumption of green leafy vegetables, consumption of fruits and juice, consumption of milk and milk products, consumption of meat and meat products, consumption of tobacco, consumption of alcohol, antenatal care, place for antenatal care (ANC), Tetanus-Diphtheria (TD) vaccination, number of iron tablets consumed, rest and sleep during pregnancy (operationally defined as whether or not the pregnant women were taking 2 h of rest during day time for current pregnancy), birth order, birth spacing, place of delivery, mode of delivery, sex of the child, birth weight. Maternal age was categorized as <20 years, 20–30 years, and > 30 years. Ethnicity was based on the caste system in Nepal and was divided into three major groups based on available literature and similarities between the caste/ethnic groups as Dalit, Aadibasi/Janajati, and the upper caste group (34). Maternal education was recorded as primary education (1–5 years of schooling), secondary education (6–10 years of schooling), and higher secondary education and above (more than 10 years of schooling). The maternal occupation was coded as non-working (household work), business/service, and agriculture/daily wage labor.

### Statistical analysis

Data were entered into Epi Data Entry 3.1 (EpiData Association, Odense, Denmark), checked for accuracy, edited as required, and then transferred to SPSS for Windows Version 27.0 (SPSS Inc., Chicago, USA) for analysis. A chi-squared test for independence was undertaken to test the associations between PTB and selected individual socio-demographic, dietary, and maternal care-related variables. The effect of each possible predictor variable on PTB was estimated using univariate analysis (crude analysis).

We used regression diagnostic procedures to for check evidence of multicollinearity or overly influential outliers in the models. However, we did not detect any multicollinearity or overly influential outliers in the models. All variables considered to be important (*p* < 0.10) were incorporated into the multi-level logistic regression analysis by backward elimination methods. Variables entered in level-I were: socio-economic variables (caste/ethnicity, family income); level-II were: socio-economic variables and food consumption behavior (caste/ethnicity, family income, consumption of additional meal, consumption of green leafy vegetables, consumption of milk and milk products); level-III were: socio-economic variables, food consumption behavior and ante-natal factors (caste/ethnicity, family income, consumption of additional meal, consumption of green leafy vegetables, consumption of milk and milk products, antenatal care, number iron tablets consumed, rest during pregnancy, birth order, and birth spacing), and level-IV were: socio-economic variables, food consumption behavior and antenatal factors and birth outcomes (caste/ethnicity, family income, consumption of additional meal, consumption of green leafy vegetables, consumption of milk and milk products, antenatal care, number iron tablets consumed, rest during pregnancy, birth order, and birth spacing, sex of child, and birth weight). Results that were significant in the final model were reported as adjusted odds ratios (AORs) with 95% confidence intervals (CI). All tests were two-tailed and *p*-values of <0.05 were considered statistically significant.

### Ethics

The study protocol was approved by the Research Ethics Committee of Janaki Medical College, Janakpur, Nepal (approval number: 11-077-078). Detailed information about the study was given to subjects before their enrollment in the study and a written informed consent was obtained thereafter. Participants' privacy, confidentiality, and anonymity were maintained in the study and personal identifiers were removed before starting data analysis.

## Results

### Personal characteristics and dietary behavior of study subjects

Tables 1, 2 show the socio-demographic characteristics of the study subjects and their dietary behavior during pregnancy. Of a total of 305 study participants, 13.77% (42/305) had preterm childbirth. The univariate analysis showed a significantly higher proportion of women who were from the Dalit caste/ethnicity (26.2%), followed by the upper caste group (12.5%) who gave birth to more preterm babies than the Aadibasi/Janajati caste/ethnicity (8.0%) (*p* = 0.003). Similarly, those subjects who were in the first tercile of the family income group (20.0%) were found to have a significantly higher proportion of preterm birth than the second and third tercile family income groups (9.8 and 9.6%, respectively, *p* = 0.039) (Table 1). Out of eight variables related to dietary behavior during pregnancy examined, only two variables were found to be statistically significant to have a higher proportion of PTB. Pregnant women who did not consume the additional meals during pregnancy had more PTB (32.5%) compared with those who consumed additional meals (10.9%), while those who did not consume green leafy vegetables gave birth to more preterm babies (32.3%) than those who consumed them (8.8%) (*p* = <0.0001) (Table 2).

**Table 1:** Socio-demographic characteristics of study subjects.

**Characteristics**	**Total** ***N* (%)**	**Preterm** ***N* (%)**	**Full-term** ***N* (%)**	***P*-value**
**Age group (in years)**				
<20 years	26 (8.5)	5 (19.2)	21 (80.8)	0.508
20-30 years	245 (80.3)	34 (13.9)	211 (86.1)	
>30 years	34 (11.1)	3 (8.8)	31 (91.2)	
**Caste/ethnicity**				
Dalit	65 (21.3)	17 (26.2)	48 (73.8)	0.003
Aadibasi/Janajati	112 (36.7)	9 (8.0)	103 (92.0)	
Upper caste	128 (42.0)	16 (12.5)	112 (87.5)	
**Religion**				
Hindu	298 (97.7)	41 (13.8)	257 (86.2)	0.968
Others	7 (2.3)	1 (14.3)	6 (85.7)	
**Education of subjects**				
Primary	86 (28.2)	15 (17.4)	71 (82.6)	0.401
Secondary	149 (48.8)	20 (13.4)	129 (86.6)	
Higher secondary	70 (23.0)	7 (10.0)	63 (90.0)	
**Education of husband**				
Primary	74 (24.3)	14 (18.9)	60 (81.1)	0.277
Secondary	137 (44.9)	15 (10.9)	122 (89.1)	
Higher secondary	94 (30.8)	13 (13.8)	81 (86.2)	
**Occupation of subjects**				
Housework	250 (82.0)	32 (12.8)	218 (87.2)	0.301
Service/business	49 (16.0)	8 (16.3)	41 (83.7)	
Agriculture/ labor	6 (2.0)	2 (33.3)	4 (66.7)	
**Occupation of husband**				
Abroad	62 (20.3)	6 (9.7)	56 (90.3)	0.497
Service/business	85 (27.9)	141 (6.5)	71 (83.5)	
Agriculture/ labor	158 (51.8)	22 (13.9)	136 (86.1)	
**Type of family**				
Joint/extended	108 (35.4)	29 (14.7)	168 (85.3)	0.515
Nuclear	197 (64.6)	13 (12.0)	95 (88.0)	
**Family income**				
1st tercile	120 (39.3)	24 (20.0)	96 (80.0)	0.039
2nd tercile	102 (33.4)	10 (9.8)	92 (90.2)	
3rd tercile	83 (27.3)	8 (9.6)	75 (90.4)	

**Table 2:** Dietary behavior during pregnancy among study subjects.

	**Total *N* (%)**	**Preterm *N* (%)**	**Full-term *N* (%)**	***P*-value**
**Nature of diet**				
Vegetarian	79 (25.9)	15 (19.0)	64 (81.0)	0.118
Non-vegetarian	226 (74.1)	27 (11.9)	199 (88.1)	
**Consumption of additional meal**				
No	40 (13.1)	13 (32.5)	27 (67.5)	<0.0001
Yes	265 (86.9)	29 (10.9)	236 (89.1)	
**Consumption of green leafy vegetables**				
No	65 (21.3)	21 (32.3)	44 (67.7)	<0.0001
Yes	240 (78.7)	21 (8.8)	219 (91.3)	
**Consumption of fruits and juice**				
No	183 (60.0)	28 (15.3)	155 (84.7)	0.342
Yes	122 (40.0)	14 (11.5)	108 (88.5)	
**Consumption of milk and milk products**				
No	204 (66.9)	33 (16.2)	171 (83.8)	0.083
Yes	101 (33.1)	9 (8.9)	92 (91.1)	
Consumption of meat and meat products*				
No	152 (49.8)	21 (13.8)	131 (86.2)	0.214
Yes	74 (24.2)	6 (8.1)	68 (91.9)	
**Consumption of tobacco**				
Yes	7 (2.3)	1 (14.3)	6 (85.7)	0.968
No	298 (97.7)	41 (13.8)	257 (86.2)	
**Consumption of alcohol**				
Yes	17 (5.6)	3 (17.6)	14 (82.4)	0.633
No	288 (94.4)	39 (13.5)	249 (86.5)	

*n = 226.

### Maternal care and newborn characteristics

Table 3 presents the results of univariate analysis on the effect of antenatal, natal, postnatal, newborn characteristics and obstetric history of pregnant women associated with PTB. Those who received <4 ANC visits (41.7%), did not consume recommended iron (<180 iron tablets) during pregnancy (22.4%), did not have adequate rest and sleep had a significantly higher rate of preterm birth (50.0%) (*p* = <0.0001) than their counterparts. In addition, our study also found that birth order first-second (16.8%) compared to third or more (8.7%) (*p* = 0.045) and birth spacing ≤ 2years (24.3%) compared to more than 2 years (11.8%) (*p* = 0.025) had significantly higher rates of PTB. Likewise, mothers who gave birth to LBW babies (28.6%) were found to have significantly higher rates of PTB than normal (11.6%) (*p* = 0.006).

**Table 3:** Antenatal, natal and postnatal care characteristics, maternal obstetric history, and newborn characteristics associated with PTB.

**Characteristics**	**Total** ***N* (%)**	**Preterm** ***N* (%)**	**Full-term** ***N* (%)**	***P*-value**
**Antenatal care**				
<4 times	36 (11.8)	15 (41.7)	21 (58.3)	<0.0001
≥4 times	269 (88.2)	27 (10.0)	242 (90.0)	
**Place for ANC**				
Primary health care center	57 (18.7)	11 (19.3)	46 (80.7)	0.395
Government hospital	123 (40.3)	16 (13.0)	107 (87.0)	
Private hospital	125 (41.0)	15 (12.0)	110 (88.0)	
**TD vaccinated**				
No	2 (0.7)	1 (50.0)	1 (50.0)	0.136
Yes	303 (99.3)	41 (13.5)	262 (86.5)	
**Number of iron tablets consumed**				
<180 tablets	143 (46.9)	32 (22.4)	111 (77.6)	<0.0001
≥180 tablets	162 (53.1)	10 (6.2)	152 (93.8)	
**Adequate rest and sleep during pregnancy**				
No	18 (5.9)	9 (50.0)	9 (50.0)	<0.0001
Yes	287 (94.1)	33 (11.5)	254 (88.5)	
**Birth order**				
First-second	190 (62.3)	32 (16.8)	158 (83.2)	0.045
Third-more	115 (37.7)	10 (8.7)	105 (91.3)	
**Birth spacing (*****n*** **=** **189)**				
≤ 2 years	70 (23.0)	17 (24.3)	53 (75.7)	0.025
More than 2 years	119 (39.0)	14 (11.8)	105 (88.2)	
**Place of childbirth**				
Home	11 (3.6)	2 (18.2)	9 (81.8)	0.665
Health facility	294 (96.4)	40 (13.6)	254 (86.4)	
**Mode of childbirth (*****n*** **=** **258)**				
Normal	156 (51.1)	20 (12.8)	136 (87.2)	0.870
Cesarean section (C/S)	8 (2.6)	1 (12.5)	7 (87.5)	
Vacuum delivery	141 (46.2)	21 (14.9)	120 (85.1)	
**Sex of child**				
Male	164 (53.8)	28 (17.1)	136 (82.9)	0.071
Female	141 (46.2)	14 (9.9)	127 (90.1)	
**Birth weight (*****n*** **=** **294)**				
Low weight (<2,500 grams)	35 (11.9)	10 (28.6)	25 (71.4)	0.006
Normal weight (≥2,500 grams)	259 (88.1)	30 (11.6)	229 (88.4)	

### Maternal characteristics, dietary behavior, antenatal care, and obstetric history associated with PTB

Table 4 shows the results of multi-level logistic regression analysis for PTB among study subjects. Maternal characteristics such as caste/ethnicity, family income, birth order, and birth spacing, dietary behavior such as consumption of additional meals, consumption of milk or milk products during pregnancy, components of antenatal care practices-ANC visits, consumption of a recommended amount of IFA (≥ 180 tablets), and rest and sleep were significantly associated with preterm birth in the final adjusted model.

**Table 4:** Factors associated with pre-term delivery by multilevel logistic regression analysis.

**Characteristics**	**COR (95%CI)**	**AOR (95%CI)**			
**(l0ptr0pt)3-6**		**Model-I**	**Model-II**	**Model-III**	**Model-IV**
**Caste/ethnicity**					
Dalit	2.47 (1.15–5.31)	4.05 (1.68–9.74)	–	14.52 (2.78–75.76)	12.16 (2.28–64.61)
Aadibasi/janajati	0.61 (0.25–1.44)	2.47 (1.15–5.31)	–	3.94 (1.02–15.15)	3.83 (1.01–14.65)
Upper caste group	Reference	Reference	–	Reference	Reference
**Family income**					
1^st^ tercile	2.34 (1.00–5.91)	–	–	6.95 (1.67–28.93)	6.82 (1.65–28.08)
2^nd^ tercile	1.01 (0.38–2.71)	–	–	2.29 (0.52–10.07)	2.25 (0.51–9.88)
3^rd^ tercile	Reference	–	–	Reference	Reference
**Consumption of additional meal**					
No	3.91 (1.82–8.42)	–	–	9.07 (2.10–39.18)	9.53 (2.13–42.55)
Yes	Reference	–	–	Reference	Reference
**Consumption of green leafy vegetables**					
No	4.97 (2.50–9.88)	–	5.80 (2.84–11.84)	–	–
Yes	Reference	–	Reference	–	–
**Consumption of milk and milk products**					
No	1.97 (0.90–4.30)	–	2.63 (1.15–6.04)	6.38 (1.56–26.07)	6.44 (1.56–26.51)
Yes	Reference	–	Reference	Reference	Reference
**Antenatal care**					
Less than 4 times	6.40 (2.95–13.86)	–	–	4.83 (1.43–16.23)	4.29 (1.25–14.67)
More than 4 times	Reference	–	–	Reference	Reference
**Number iron tablets consumed**					
<180 tablets	4.38 (2.06–9.28)	–	–	3.35 (1.00–11.28)	3.46 (1.03–11.58)
≥ 180 tablets	Reference	–	–	Reference	Reference
**Rest during Pregnancy**					
No	7.69 (2.85–20.76)	–	–	5.08 (1.05–24.40)	4.83 (1.01–23.30)
Yes	Reference	–	–	Reference	Reference
**Birth order**					
First-second	2.12 (1.01– 4.51)	–	–	9.29 (1.69–50.91)	9.56 (1.74–52.53)
Third-more	Reference	–	–	Reference	Reference
**Birth spacing**					
≤ 2	2.40 (1.10–5.25)	–	–	5.41 (1.71–17.11)	5.16 (1.62–16.42)
More than two	Reference	–	–	Reference	Reference
**Sex of child**					
Male	1.86 (0.94–3.70)	–	–	–	–
Female	Reference	–	–	–	–
**Birth weight**					
Low weight	3.05 (1.33–6.97)	–	–	–	–
Normal weight	Reference	–	–	–	–

AOR, adjusted odds ratio; COR, crude odds ratio; CI, confidence interval; All variables considered to be important (p <0.10) in univariate analysis were entered into the multi-level logistic regression analysis.

Mothers from Dalit caste/ethnicity (AOR = 12.16, 95% CI = 2.28–64.61) and Aadibasi/Janajati caste/ethnicity (AOR = 3.83, 95% CI = 1.01–14.65) compared to upper caste group, family income in the first tercile (AOR = 6.82, 95% CI = 1.65–28.080) than those in third tercile were more likely to give preterm birth. Likewise, birth order first-second (AOR = 9.56, 95% CI = 1.74–52.53) than third or more, and birth spacing ≤ 2 years (AOR = 5.16, 95% CI = 1.62–16.42) were at greater risk of PTB. Mothers who did not consumed additional meal (AOR = 9.53, 95% CI = 2.13–42.55), milk and milk products (AOR = 6.44, 95% CI = 1.56–26.51) during pregnancy, having <4 times ANC visits (AOR = 4.29, 95% CI = 1.25–14.67), not having the recommended amount of IFA intake (<180 tablets) (AOR = 3.46, 95% CI = 1.03–11.58), and not having adequate rest and sleep (AOR = 4.83, 95% CI = 1.01–23.30) during pregnancy had higher odds of having PTB than their counterparts.

## Discussion

In this community-based cross-sectional study, of total of 305 mother-live-born baby pairs of <6 months, we found that 13.77% (42/305) had preterm childbirth. Some sociodemographic factors such as mothers from Dalit and Aadibasi/Janajati caste/ethnicity, family income in the first tercile, and maternal factors such as first-second birth spacing ≤ 2 years, having <4 antenatal (ANC) visits, not having the recommended amount of IFA intake (<180 tablets), not having adequate rest and sleep during pregnancy, had higher odds of having PTB. In addition, we found some maternal dietary behavior-related factors such as mothers who did not consume additional meals, milk and milk products during pregnancy, were positively associated with PTB.

PTB is disproportionately concentrated in low-income countries in Asia and Africa. It has been estimated that about 11 million (85%) PTB occurred in Asia and Africa compared to around 0.5 million PTB in Europe and North America together (excluding Mexico) in the year 2005 (35). There are different factors that are associated with PTB in published literature, such as the sociodemographic, geographic, pre-existing, and existing medical and obstetrical conditions and other attributes of the subjects (17, 20, 24). The proportion of PTB in our study (13.77%), however, is comparable to previous studies from low-and-middle income countries being conducted in Tanzania (17), and Kenya (18), while it is lower than that of the reports from Bangladesh (22.3%) (21). Studies being conducted in different geographical settings of Nepal reported PTB ranged from 9.8 to 21.2% between 2014 and 2020 (25, 26, 36) suggesting a continued burden of PBT in Nepal.

In this study, we observed some sociodemographic factors such as mothers who were from Dalit, and Aadibasi/Janajati caste/ethnicity and family income in the first tercile were more likely to have PTB. The caste-based system and lower family economy of Nepal have an inverse relation to health and well-being including maternal health (37). Published literature has demonstrated several maternal health issues such as a higher proportion of teenage pregnancy (38), and lower utilization of available maternal health care services (39) in disadvantaged caste/ethnic groups (Dalit and Aadibasi/Janajati). Specific health measures in Nepal should, therefore, be targeted to those vulnerable caste/ethnic groups, and those who are in lower economic strata to improve the burden of PTB.

Similar to the previous reports from other low-and-middle-income countries (21, 23, 40), we also found maternal factors such as birth order first-second, birth spacing ≤ 2 years, having <4 antenatal (ANC) visits, not having the recommended amount of IFA intake (<180 tablets), not having adequate rest and sleep during pregnancy had higher odds of having PTB. Since very few studies from Nepal reported such predictors of PTB, we recommend further studies to confirm such findings.

Interestingly, we found some maternal dietary behavior-related factors such as mothers who did not consume additional meals, milk and milk products during pregnancy were positively associated with PTB. Pregnant women are encouraged to take adequate dietary constituents during pregnancy to meet the increased physiological requirements for themselves and their babies. Nonetheless, the exact mechanism of whether adequate dietary intake with the optimum mix of essential dietary constituents consumed by pregnant women will reduce the likelihood of PTB, and the impact of dietary intervention during pregnancy seems inconclusive whether reduces PTB. While there is some convincing evidence of the beneficial impacts of dietary (reduced probability of cesarean sections, and gestational diabetes), and physical activity intervention during pregnancy, there are variable reports of such interventions (41–43). As such, a meta-analysis conducted by Rogozińska et al. (41) demonstrated no significant effect of the diet and lifestyle intervention on PTB whereas a prospective cohort study from Denmark showed maternal intake of a Mediterranean-type diet (MD) reduced PTB (43). Additional studies might therefore be necessary to validate such results.

Given that there is limited research in Nepal that examines the predictors of PTB, this study is important to narrow down the literature gap and provide new insights that might be useful to formulate and implement evidence-based health strategies in the current study setting and other similar settings. There are some important strengths of the study to mention: First, this study has a high response rate (94%). Second, it used study instruments adapted from the NDHS 2016. However, this study had some specific limitations that had to be considered while interpreting the results. First, this study did not examine many other known risk factors (maternal obstetrical and medical conditions) and some other dietary factors as the data were unavailable. Second, this study was cross-sectional, which limits the possibility to establish a causal association between exposure and outcome. Third, the study setting and sample size were relatively small, which reduced the external validity. Furthermore, we used the Cochran formula (31) to calculate the sample size although the primary aim was to determine the risk factors of PTB and not PTB prevalence. Finally, self-reported results might have been influenced by recall bias although we collected the specific information from maternity service registers. However, local area studies have their own importance in terms of local area-specific customized health strategy formulation to reduce the burden of health problems. We recommend additional follow-up studies be conducted to validate the study outcomes.

## Conclusion

This study found that some socio-demographic (Dalit and Aadibasi/Janajati caste/ethnicity, family income in the first tercile), maternal (birth order third, birth spacing, <4 antenatal ANC visits, intake of the recommended amount of IFA, not having adequate rest and sleep during pregnancy) and dietary behavior related factors (mothers who did not consume additional meals, and milk and milk products during pregnancy) were independently associated with PTB. In the current setting of Southeastern Nepal, health promotion strategies should target disadvantaged groups (Dalit and Aadibasi/Janajati caste/ethnicity and those economically deprived). In addition, it is recommended to have some specific measures such as promoting antenatal mothers to use the available antenatal care services and counsel to have an adequate diet to a level that meets their daily requirements. Additional studies are needed to explore the obstetrical, medical, and additional dietary factors associated with PTB in Nepal.

## Data availability statement

The raw data supporting the conclusions of this article will be made available by the authors, without undue reservation.

## Ethics statement

The studies involving human participants were reviewed and approved by Research Ethics Committee of Janaki Medical College, Janakpur, Nepal (Approval Number: 11-077-078). The patients/participants provided their written informed consent to participate in this study.

## Author contributions

DA, SG, and JS: conceptualized the study, performed investigation, data collection, formal analysis, and writing—original draft preparation. TGP, AL, AG, and KL: formal analysis, review and editing, and writing—review and editing. All authors contributed to the article and approved the submitted version.

## Conflict of interest

The authors declare that the research was conducted in the absence of any commercial or financial relationships that could be construed as a potential conflict of interest.

## Publisher's note

All claims expressed in this article are solely those of the authors and do not necessarily represent those of their affiliated organizations, or those of the publisher, the editors and the reviewers. Any product that may be evaluated in this article, or claim that may be made by its manufacturer, is not guaranteed or endorsed by the publisher.
